# Improved Removal of Neonicotinoid Insecticides from Real Water Matrices by Modified UF and NF Membranes

**DOI:** 10.3390/membranes16090289

**Published:** 2026-08-28

**Authors:** Francisco J. Real, Juan L. Acero, Esther Matamoros, Carolina Godoy

**Affiliations:** Departamento de Ingeniería Química y Química Física, Instituto Universitario de Investigación del Agua, Cambio Climático y Sostenibilidad (IACYS), Facultad de Ciencias, Universidad de Extremadura, Avda. de Elvas s/n, 06006 Badajoz, Spain; jlacero@unex.es (J.L.A.); esthermc@unex.es (E.M.); carolinagr@unex.es (C.G.)

**Keywords:** neonicotinoids, membrane technologies, ultrafiltration, nanofiltration, modified membranes, WWTP effluents

## Abstract

The removal of five neonicotinoid insecticides, acetamiprid, chlothianidin, imidacloprid, thiacloprid, and thiamethoxam, was explored using various commercial ultrafiltration (MW, PT, and GK) and nanofiltration (HL) membranes provided by GE Osmonics Labstore. Several modification techniques have also been applied to one ultrafiltration membrane, including immersion in hot water, sodium hydroxide, and ethanol solutions, as well as interfacial polymerization with monomers such as polyethyleneimine and trimesoyl chloride, to improve micropollutant retention while maintaining adequate permeability. The results show that only immersion in ethanol (60% solution or absolute ethanol) improved membrane performance. Among the reagents and conditions tested for membrane surface modification via polymerization, the sequential application of polyethyleneimine, trimesoyl chloride, oven curing at 60 °C, followed by immersion in a glycerol solution was the most efficient. The optimal modified membrane was tested with real water matrices (two secondary effluents from wastewater treatment plants and a surface water sample) in which the neonicotinoids were dissolved. The modified ultrafiltration membrane showed higher retention (80–95%) than commercial ultrafiltration (15–60%) and levels similar to nanofiltration membranes (70–95%), demonstrating greater efficiency in retaining neonicotinoids under real water conditions, although the permeability was about half that of the commercial nanofiltration membrane. Therefore, this modification process is a promising alternative to commercial membranes for removing micropollutants from urban wastewater and should be considered.

## 1. Introduction

Neonicotinoids are systemic pesticides widely used in agriculture to control harmful insects because they are more toxic to invertebrates than to mammals and birds. However, their use as insecticides has raised concerns about harmful effects on bees and even humans [1,2,3] because of their environmental persistence and high water solubility. Neonicotinoids have been found in low concentrations in soils, water, and food [4]. Although the use of most neonicotinoids as pesticides is now more restricted due to their negative effects on pollinating species, they are still found in the environment, including surface water and wastewater, through the food chain [5,6,7]. Long-term environmental exposure to neonicotinoids, even at very low concentrations, may pose risks to humans and other mammals because they can disrupt physiological functions [2,8].

Because most micropollutants, including neonicotinoids, are not efficiently removed by conventional wastewater treatment plants (WWTPs), the European Union recently adopted Directive (EU) 2024/3019 [9], which establishes a framework for implementing quaternary treatment technologies to efficiently remove poorly biodegradable micropollutants from WWTP effluents, achieving 80% average removal of selected organic micropollutants. The main technologies for removing these micropollutants are chemical oxidation processes, including ozonation and combinations with other agents through advanced oxidation processes, as well as physical filtration techniques using activated carbon and membranes [10,11].

Among membrane processes for quaternary treatment, reverse osmosis (RO) and nanofiltration (NF) are efficient techniques, as these offer retention levels that approach the minimum of 80% required by the European directive [10,12]. Although ultrafiltration (UF) is not considered a quaternary treatment on its own due to its low retention levels, it can be used as a pretreatment for NF and RO. Furthermore, several hybrid technologies have gained importance as promising solutions for removing these micropollutants from water [13,14], as they improve membrane properties such as hydrophilicity, fouling resistance, permeability, and mechanical stability. Among these, adsorption-membrane hybrid technologies, magnetic nanocomposites, and membrane modification stand out [15]. In the last decade, considerable research has focused on modifying commercial UF membranes to improve their retention performance. Among the modification techniques tested, two main types of procedures have proven successful: membrane immersion in various solutions that induce property changes, and interfacial polymerization, which forms a layer on the membrane to improve retention capacity. In the first technique, researchers tested different solutions and immersion conditions. Thus, Ji et al. [16] explored the improvements and drawbacks experienced by membranes after pretreatment by immersion in hot water, ethanol, and NaOH solutions, processes previously reported to produce structural changes, such as pore enlargement in polysulfone membranes. They observed a significant improvement in membrane permeability, especially with ethanol, as well as increased adsorption retention. As for the second technique, researchers have studied membrane modification more extensively [17,18,19], using monomers such as polyethyleneimine (PEI), trimesoyl chloride (TMC), polyethylene glycol, or polyvinylpyrrolidone. Several investigations showed that sequentially applying PEI and TMC monomers to a polysulfone or polyamide membrane resulted in instantaneous polymerization through cross-linking between the two monomers [19,20]. Yu et al. [21] studied crosslinked UF membrane polymerization using PEI and phenolphthalein-based poly(ether ether ketone), which improved dye retention, antifouling performance, and even water permeability. Also, Li et al. [22] cross-linked a UF membrane with PEI and carboxylic acid, which were introduced into the inner pores, achieving a pore size of 1.2 nm on the NF scale, and leading to excellent dye retention (99.9% for Congo red, MW = 697 g/mol), antifouling performance, hydrophilicity, and long-term operational stability. Therefore, PEI was one of the most widely used monomers in these studies to form a polymer layer on the membrane surface, and it was selected for this work.

Previous studies have removed the neonicotinoids under study through chemical oxidation processes, considered as quaternary treatment, including combinations of ozonation with adsorption onto activated carbon [23,24]. However, filtration techniques have been applied to neonicotinoids very rarely. Therefore, this study has focused on the removal of the five neonicotinoids (acetamiprid (ACE), chlothianidin (CLO), imidacloprid (IMI), thiacloprid (THC), and thiamethoxam (THM)) using UF and NF membranes made primarily of polysulfone or polysulfone/polyamide composite materials. Special emphasis will be placed on the preparation of modified membranes using immersion and interfacial polymerization techniques, employing the most effective chemical agents: hot water, ethanol, and NaOH solutions for the immersion technique, and the combined use of PEI and TMC monomers, along with the surfactant SDS, for the interfacial polymerization technique. The filtration efficiency of these modified membranes will be evaluated in ultrapure water, surface water, and WWTP effluents. The main objectives were to study the performance of different types of membranes, as well as to try to improve their behavior by modifying the surface of UF membranes, to achieve the average removal levels of 80% required for neonicotinoids, taken as an example of organic contaminants, as well as to investigate the filtration process of real water matrices containing these contaminants.

## 2. Materials and Methods

### 2.1. Reagents, Membranes, and Water Matrices Used

Neonicotinoids ACE, CLO, IMI, THC, and THM were purchased from Sigma-Aldrich (Madrid, Spain) of the highest available purity. Table 1 compiles their main physicochemical properties. PEI ((C_2_H_5_N)_n_) and TMC (C_9_H_3_Cl_3_O_3_), used to modify the surface of UF membranes, were also obtained from Sigma-Aldrich (Spain). The surfactant sodium dodecyl sulfate (SDS), used to alter membrane hydrophilicity and promote interfacial polymerization, was purchased from Scharlab S.L. (Senmenat, Spain). The remaining general reagents were purchased from Panreac S.L.U. (Castellar del Vallès, Spain).

Four different membranes purchased from GE Osmonics Labstore (FL, USA) were used in this research: the UF membranes denoted as MW, PT, and GK, made of polyacrylonitrile (PAN), polyethersulfone (PES), and polysulfone-polyamide thin-film composite (TF), respectively; and the NF membrane HL, also made of TF. The main properties of these filtration membranes are shown in Table 2.

Ultrapure (UP) water to prepare the solutions was provided by a Milli-Q water system (Millipore, MA, USA). Three water matrices (surface water (SW) and two secondary effluents from WWTPs (SE1 and SE2)) were collected in the Extremadura Community (Southwest of Spain). Table 3 compiles their main quality parameters.

### 2.2. Experimental Procedures

The UF and NF filtration experiments were carried out in the laboratory cross-flow membrane filtration unit model P-28, supplied by CM-CELFA Membrantechnik AG (Seewen, Switzerland), as described in previous research [25,26]. Briefly, it consisted of a 500 mL pressurized storage vessel with a device containing the membrane. The effective surface area (A) was 28 cm^2^. The transmembrane pressure (TMP) varied from 2 to 20 bar depending on the membrane used in each experiment. We controlled TMP by feeding nitrogen gas to the head of the storage vessel. The temperature and tangential velocity (v) were kept constant at 20 °C and 2 m/s, respectively. We used a new membrane for each experiment.

The experiments were carried out in batch concentration mode, with the permeate stream collected separately and the retentate stream recycled to the feed tank. Before starting each experiment, the UP water permeate flux (J_w1_) was measured (Equation (1)) to determine the pure-water permeability (PWP, J_w1_/TMP). Then, the filtration experiment began after adding an initial volume (V_0_) of 300 mL of the water matrix containing 1 μM of individual neonicotinoids (C_0i_). At regular time intervals (t), the permeate flux (J_v_) was determined from the permeate volume (V_P_), which was measured by weighing the permeate on an analytical balance. Also, some permeate samples were taken to determine the remaining content of each neonicotinoid (C_Pi_), as well as the retention percentage (Equation (2)).

(1) $$J_{W}{\;\mathrm{or}\;J}_{v}=\frac{V_{P}}{A\cdot t}$$

(2) $$\mathrm{Retention}\;\mathrm{percentage}=\frac{C_{0_{i}}-C_{P_{i}}}{C_{0_{i}}}\cdot 100$$

The experiments stopped when the volume reduction factor (VRF, Equation (3)) reached 3, yielding permeate and retentate volumes of V_P_ = 200 mL and V_R_ = 100 mL, respectively, which were stored for analysis.

(3) $$\mathrm{VRF}=\frac{V_{0}}{V_{0}-V_{P}}=\frac{V_{0}}{V_{R}}$$

After each experiment, the membrane was rinsed with UP water for 5–10 min to remove external fouling. The UP water permeate flux was then determined again (J_w2_) to assess irreversible membrane fouling (internal fouling) as a function of membrane recovery (Equation (4)). The adsorption percentages of each neonicotinoid on the membrane surface were also determined from the volume and concentrations in each fraction (Equation (5)). We also performed blank tests and suitable replicates for experiments with real water matrices.

(4) $$\mathrm{Membrane}\;\mathrm{recovery}=\frac{J_{w_{2}}}{J_{w_{1}}}\cdot 100$$

(5) $$\mathrm{Adsorption}\;\mathrm{percentage}=\frac{V_{0}\cdot C_{0_{i}}-V_{P}\cdot C_{P_{i}}-V_{R}\cdot C_{R_{i}}}{V_{0}\cdot C_{0_{i}}}\cdot 100$$

In concentration mode, the retentate concentration increases unless adsorption is the sole cause of compound removal in the permeate. This will lead to an apparent decrease in the removal percentage as the VRF increases, since the percentage is referenced to the feed concentration at the start of the experiment (Equation (2)). However, we used this parameter at VRF 3 for comparison, and in the final studies we expanded it to determine the overall retention percentage using the permeate concentrations of each neonicotinoid measured in the entire permeate mass obtained (200 mL).

### 2.3. Procedure for the Preparation of Modified Membranes

The membranes were modified by two different techniques: immersion and polymerization. This first technique is based on the study by Ji et al. [16] and focuses on modifying the PT membrane through immersion, agitation, heating, and storage time in UP water. We selected three substances for this modification: water, sodium hydroxide, and ethanol. All experiments in this set used immersion in water at 70 °C for 90 min with moderate agitation as the basis. We then performed subsequent modifications with the other agents (1 M NaOH, absolute ethanol, and 60% ethanol in UP water). Some membranes were only pretreated with hot water. For 1 M NaOH and absolute ethanol, the temperature was 50 °C for 120 min, while for 60% ethanol solution, it was 35 °C for 75 min. In addition, we selected different storage times because, after surface treatment, the membrane must be rinsed with UP water several times and stored in it for at least 24 h before experimentation. Storage times of between 1 and 7 days were used. The modified PT membrane was used at a TMP of 4 bar.

The polymerization process used to prepare the modified PT membrane is based on the work of Ang et al. [19]. The membrane was wetted three times in water to moisten and wash it. It was then brought into contact with a polyethyleneimine (PEI) solution (usually, 0.8% wt%). The PEI solution was applied exclusively to the active layer using an agitated cell for 5–10 min to promote a more homogeneous coating. The surfactant sodium dodecyl sulfate (SDS, 0.05% *w*/*w*) was applied to some membranes to assess its effect on the surface. After polymerization, the membrane was dried with pressurized air and then placed back in the agitated cell for 30 s with a trimesoyl chloride (TMC) solution (0.2% *w*/*w* in hexane). After this time, it was cured in an oven at 60 °C for 5–10 min. The membrane was then immersed in a 15% glycerol (GLY) solution (*w*/*w*) for 4 h. After that, the membrane was removed from the solution and left to air dry overnight. Before use, it was immersed in UP water for one day. The TMP in the filtration experiments with these membranes varied from 4 to 30 bar.

### 2.4. Analytical Methods

The concentration of each neonicotinoid was determined by HPLC, following the analytical methods described in previous work [24,27] and in Text S1. An initial concentration of 1 µM was considered appropriate to allow accurate monitoring of each neonicotinoid in the permeate effluent mixture, given the limit of quantification of 0.02 µM. The characterization of real water matrices was carried out following the Standard Methods [28]. Further details regarding water characterization are also described in Text S1.

## 3. Results and Discussion

### 3.1. Removal of Neonicotinoids by Virgin UF and NF Membranes in Different Water Matrices

Filtration experiments were carried out with an initial concentration of 1 µM for each of the five neonicotinoids dissolved in different water matrices using three UF membranes (denoted MW, PT, and GK) and one NF membrane (denoted HL).

As a first step, we performed filtration experiments with the five neonicotinoids dissolved in UP water. Table 4 summarizes the results obtained with the four commercial membranes. Permeability studies were conducted, and the degree of fouling for each membrane used was analyzed. As can be deduced from the permeate flow values at the end of the experiments (J_vss_) exposed in Table 4, no apparent fouling of the membranes was observed since, in all cases, complete recovery of membrane permeability was verified after surface cleaning, demonstrating the absence of internal fouling (despite micropollutant adsorption).

The retention and adsorption percentages of each neonicotinoid were also evaluated according to Equations (2) and (5), respectively, and different micropollutant retention mechanisms can be proposed. The results (Table 4) clearly show that the HL membrane was the most efficient in micropollutant retention, followed by the PT membrane. Retention of neonicotinoids on the MW membrane was low, likely due to its high MWCO (50,000 Da), so adsorption was the only micropollutant retention mechanism. The PT membrane (MWCO of 5000 Da) exhibited moderate retention of neonicotinoids, mainly due to the adsorption mechanism. Moreover, the PT membrane had the highest adsorption capacity of the four membranes tested, likely due to its greater hydrophobicity (see contact angles in Table 2). The GK membrane also exhibited low micropollutant retention despite its small MWCO (2000 Da), meaning that even this UF membrane with the smallest pore size was unable to retain micropollutants through size exclusion. Furthermore, the GK membrane presented reduced micropollutant retention by adsorption onto its surface, probably due to its greater hydrophilicity.

The MW membrane, with low retention values, showed that the percentage of compound removal was practically the same for the five neonicotinoids, and the adsorption levels were also very similar. The PT membrane, which achieved higher removal percentages, showed retention values for the five neonicotinoids that fairly coincide with those of adsorption. THC had the highest removal percentage at almost 50%, followed by IMI, CLO, ACE, and THM. Similar results were observed for the GK membrane, where the retention and adsorption order was THC > CLO > IMI > THM > ACE. THC, the most retained compound, has the second-highest log K_OW_ value, implying higher hydrophobicity and, consequently, greater adsorption. THM, one of the least retained compounds, had the lowest log K_OW_ and exhibited the least hydrophobic character. However, ACE, which had one of the lowest retention percentages, had the highest log K_OW_ value. Therefore, the three UF membranes exhibited a similar order of micropollutant retention and adsorption. Furthermore, the nearly identical retention and adsorption percentages indicate that neonicotinoid retention was primarily due to adsorption. The absence of internal fouling, as previously established, suggests adsorption on the membrane surface.

The retention order for the HL membrane was THM > ACE > THC > IMI > CLO, while the adsorption order was THC > IMI > CLO > ACE > THM. Furthermore, retention values were much higher than adsorption percentages, indicating that adsorption was not the primary factor driving micropollutant removal. The most retained compound, THM, has the highest MW (291.71), followed by THC and IMI, which have very similar MW values (252.73 and 255.67, respectively). The least retained compound, CLO, has an MW of 249.68. ACE was the second most retained neonicotinoid and has the lowest MW (222.68); however, ACE has the highest molar volume. Therefore, the HL membrane retains neonicotinoids mainly through size exclusion, and the UF membranes (MW, PT, and GK) retain them by adsorption onto the membrane surface. Previous studies using these membrane types to remove different micropollutants observed similar tendencies [25,29].

Additional filtration experiments of the five neonicotinoids were performed by varying the pH of the UP water, initially at pH 6. The pH was adjusted to values of 7 and 9 by adding NaOH. The membranes with the highest micropollutant separation efficiency, PT (UF) and HL (NF), were used in these experiments. Table S1 in the Supplementary Materials shows the results. These results indicate that increasing pH had a slight effect on membrane permeability, with only a very small decrease in J_vss_ values. Membrane fouling was negligible, with 100% recovery achieved after washing in almost all cases. Neonicotinoid removal decreased for the UF PT membrane as pH increased, although values at pH 7 and 9 were similar. The decrease in retention with increasing pH was more pronounced with the NF HL membrane, especially at pH 9. The adsorption results for the PT membrane indicate that adsorption percentage decreased as pH increased. In contrast, the HL membrane showed a slight increase in adsorption as pH increased to 7, followed by a decrease or stabilization at pH 9. Typically, increasing pH increases pollutant retention in membrane filtration due to electrostatic repulsion [30]. However, since neonicotinoids are neutral compounds (Table 1), this effect was not observed. The decrease in retention with pH observed in the HL membrane suggests that pH 6–7 should be used to maintain proper NF efficiency.

Next, filtration experiments were carried out with membranes PT and HL by dissolving the neonicotinoids in the water matrices mentioned in Section 2.1: one surface water (SW) and two secondary effluents (SE1 and SE2). These experiments aimed to evaluate the influence of the water matrix on the retention coefficients of each pollutant and to analyze changes in water quality parameters, such as total nitrogen and total phosphorus, absorbance at 254 nm (A_254nm_), and dissolved organic carbon (DOC). Table S2 summarizes the results on permeability, retention, and adsorption of the neonicotinoids obtained in these experiments. These results show little membrane fouling across different water matrices (J_vss_/J_w1_ close to 1), and permeability recovered to near 100% after washing. Regarding neonicotinoid reduction in the permeate stream, PT membrane efficiency decreased significantly when water matrices other than UP water were used. This decrease may be associated with a similar reduction in neonicotinoid adsorption on the membrane. Neonicotinoids likely compete with components of the real water samples for adsorption onto the membrane surface, limiting neonicotinoid adsorption. In fact, neonicotinoid retention and adsorption decreased most with SE2 effluent, which had the highest organic matter content. The removal of neonicotinoids by the HL membrane was only slightly reduced in secondary effluents, while adsorption remained the same or even increased slightly, especially in the experiment with SE1, which had the highest concentration of ionic species and the lowest content of organic matter. The order of retention of the neonicotinoids in real waters was very similar to that observed in UP water, with THC being the most retained in the PT membrane and THM the least, as also occurred with the adsorption levels; while THM was the most retained and CLO the least with the HL membrane, regardless of the water matrix used.

Table S3 shows the removal percentages of water pollution parameters in these experiments. As expected, the removal percentages of A_254nm_, turbidity, total nitrogen, total phosphorus, and DOC were higher with the HL membrane than with the PT membrane. A_254nm_ and DOC decreased by more than 85 and 76%, respectively, with the HL membrane, while the PT membrane showed much lower removals. Retention was lower in the SE1 effluent, likely due to its low initial pollutant load. Therefore, NF treatment with the HL membrane produced a final effluent, or reclaimed water, of good quality for reuse in several applications. Because the final effluent from UF treatment with the PT membrane was poor quality, we investigated modifying the PT membrane surface to obtain reclaimed water of good quality for reuse.

### 3.2. Removal of Neonicotinoids Using Modified UF Membranes: Testing Different Modification Techniques in UP Water

After completing the filtration experiments with the original commercial membranes, several modification tests were performed on the PT membrane using two techniques, immersion and interfacial polymerization, according to the procedures described in Text S1.

In the first stage, membrane modification tests by immersion in hot water (70 °C for 90 min) were performed. In addition, some experiments were carried out by subsequently immersing the membranes in 1 M NaOH solution, absolute ethanol, or 60% ethanol solution. Afterward, the membranes were stored in cold water for different periods. Table 5 summarizes the results of the experiments. The membrane permeate flux values improved with the modifications, especially after immersion in NaOH and ethanol, increasing J_vss_ from 100 to about 160 L/(h m^2^). This general increase in permeate flux can be explained by the partial removal of preservative reagents on the membrane surface, such as polyvinylpyrrolidone (PVP), one of the most common additives added during membrane manufacturing to form pores [16]. Furthermore, NaOH treatment hydrolyzes the lactam ring of PVP, generating additional hydrophilic functional groups that enhance membrane permeability [31]. Ethanol pretreatment yielded the highest permeability values, which can be explained by two mechanisms: the previously described removal of preservatives from the membrane surface and swelling of the outermost membrane layer due to ethanol interactions [32]. Both mechanisms change the membrane’s composition and structure, such as increasing pore size. Results at different storage times (1 and 7 days) after the membrane was modified with NaOH and absolute ethanol were very similar. Therefore, subsequent experiments tested only a 1-day storage time using a 60% ethanol solution as the immersion reagent. Different ethanol concentrations did not appear to significantly affect membrane permeability, although some studies reported higher permeability with absolute ethanol than with 60% solutions [16]. Based on the results, surface modifications with NaOH and ethanol were more effective at improving permeability than pretreatment with hot water alone, and storage time after modification did not affect the membranes.

However, neonicotinoid removal percentages were lower in experiments with PT membranes modified with hot water and 1 M NaOH than with the virgin PT membrane. It is also worth noting that the retention percentages of the five pollutants in the experiments with hot water and 1 M NaOH pretreatment were very low, ranging from 9.5% to 14.1%, except for THC, which ranged from 16% to 21.4%, while in the experiment performed without pretreatment, the retention values were moderate, ranging from 22.6% to 51.3% for THM and THC, respectively. Based on these findings, further work with hot water and NaOH is not recommended. However, in experiments with ethanol modification, neonicotinoid retention was considerably higher than with the virgin membrane. Conversely, after pretreatment, pollutant retention decreased slightly with storage time (from 1 to 7 days), suggesting a negative effect of storage time on retention. Finally, in the experiment conducted with a 60% ethanol solution, a somewhat less significant increase in pollutant retention (compared to absolute ethanol experiments) was observed. In general, adsorption percentages were very similar to retention percentages, indicating that the primary retention mechanism remains adsorption onto the membrane surface. In conclusion, in this set of experiments, the best modification is immersion in ethanol solutions, as it improves both permeability and retention capacity by adsorption onto the membrane while reducing operating time. However, the high levels of adsorption, the main retention mechanism, make it difficult to use this modified membrane for long periods, requiring more frequent stops for membrane cleaning.

In a second stage, interfacial polymerization techniques were applied following the procedure described in Text S1 to determine the best membrane efficiency in terms of permeability and micropollutant retention. Several experiments were conducted adding two monomers, PEI and TMC, although only PEI was used in some experiments; in others, the surfactant SDS was also added, as described in Text S1. After these pretreatments, the membrane was cured in an oven at 60 °C, and additional modifications (such as immersion in GLY solution) were also considered. Table S4 summarizes results from experiments conducted with neonicotinoids dissolved in ultrapure (UP) water, in which the reagents used for membrane modification via polymerization were varied, while oven curing was the sole post-treatment step. The first consequence of using these modified membranes, compared to the virgin one, was a decrease in PWP by around an order of magnitude, regardless of the number of reagents used. This led to a substantial increase in the time required to reach previous filtration levels (VRF 3), even when the TMP was raised from 6 to 30 bar in some experiments. Therefore, Table S4 shows neonicotinoid retention when only a small volume of permeate was produced, corresponding to a VRF of 1.1 (VP = 27.3 mL versus 300 mL of feed). Table S4 also summarizes the permeate fluxes obtained in these experiments. A decrease in permeate flux was observed throughout the experiment (J_vss_ < J_w1_), indicating slight fouling, which was scarcely recovered after washing (J_w2_ < J_w1_). This suggests some internal fouling within the membrane pores, as well as external fouling on its surface.

Regarding the neonicotinoid retention data from this set of experiments, elimination of these compounds increased markedly compared with the blank experiment using the virgin membrane. In some experiments, retention values of approximately 90% were achieved for all compounds, whereas the virgin membrane exhibited retention values ranging from 17% to 38%. These results, both the retention of micropollutants and the decrease in permeability, are likely because the membrane transitioned from the UF to the NF range. Surface polymerization reduced pore size, shifting the filtration range. Among the experiments with membranes modified with different agents, the PEI+TMC combination achieved the highest retention levels. Membranes modified solely with PEI showed lower retention levels, while SDS showed no improvement; therefore, these modification methods are ruled out, despite previous studies [19] reporting that adding SDS was favorable. These results suggest that TMC contributes to membrane surface modification by cross-linking PEI chains. This leads to reduced permeability and increased pollutant retention due to the increased membrane surface density. Therefore, the best option among those investigated so far is the treatment with PEI+TMC+60 °C, since it allows the construction of a network of chains between the base membrane, the PEI polymer, and the TMC, creating a new NF membrane, which presents the best data on micropollutant retention in the first stages of the filtration process, assuming that the retention trend will be similar over time. Consequently, the modification procedure using PEI and TMC monomers followed by heat treatment at 60 °C was applied in subsequent stages of the research.

As previously mentioned, crosslinking between the PEI and TMC chains is expected to cause membrane densification and a change in pore size, as reported in other studies [18]. This densification process is largely beneficial, but if the membrane becomes too dense, the pore size can become so small that it becomes detrimental to the filtration process [20]. Therefore, a new variable was introduced: immersing the modified membrane in a 15% glycerol (GLY) solution (15 wt%) after curing at 60 °C for 10 min. This stops the crosslinking process before the membrane collapses. Furthermore, because the first part of Section 3.2 demonstrated ethanol’s effectiveness in removing preservative residues from the membrane surface and its ability to increase hydrophilicity and pore size, thereby improving permeability, we investigated the effect of combining this ethanol immersion pretreatment with the subsequent polymerization treatment. Table 6 summarizes the results obtained with modified membranes following these procedures.

Table 6 shows that when the polymerized membrane was immersed in a GLY solution for 4 h, permeability decreased, and the TMP had to be increased from 4 bar (using the virgin PT membrane) to 20 bar. However, compared with complete treatment without stopping the densification process, the permeate flux improved, the TMP could be reduced from 30 to 20 bar, and the experiment could be completed to VRF 3. Therefore, post-treatment with glycerol effectively reduced membrane densification. This process increased neonicotinoid retention and adsorption compared to the virgin membrane. The retention levels at VRF 3 were lower than those achieved at VRF 1.1 due to increasing neonicotinoid concentrations in the retentate stream, which was recirculated, while its volume, V_R_, decreased. This decrease did not occur in the blank experiment, since when adsorption was the primary retention mechanism, pollutant concentration did not increase in the recirculated solution [33]. This reduction in the retention levels with increasing VRF also implies that the overall retention value in the permeate volume at VRF 3 (V_P_ = 200 mL) was greater than the specific retention measured at that time. Regarding the ethanol pretreatment, Table 6 shows that the TMP could be reduced to 8 bar, and the permeate flux decreased steadily as filtration progressed, falling at a VRF of 3 (J_vss_) to below 40% of the initial value. This indicates that the membrane collapsed over time. At the same time, neonicotinoid retention and adsorption did not improve compared with experiments without this pretreatment. Therefore, ethanol pretreatment was not very suitable for improving the efficiency of modified membranes.

Finally, the effect of curing time at 60 °C (5 and 8 min) was investigated, as well as the TMP applied in each experiment (between 8 and 15 bar) for membranes modified by interfacial polymerization using PEI+TMC+60 °C+GLY. Table S5 summarizes the results obtained in this set of experiments. The first objective was to determine the minimum oven curing time. Reducing the oven curing time decreased membrane densification and, therefore, chain crosslinking, resulting in a larger pore size. However, the ideal pore size is not necessarily the smallest, as excessively small pores can also cause problems with filtration development. If the pore size is too small, permeability decreases to the point where the process is no longer viable [20]. According to Table S5 results for experiments performed at a TMP of 10 bar, decreasing the curing time from 8 to 5 min increased membrane permeability. At the same time, neonicotinoid retention was slightly higher in the experiment with 5 min of curing time. Preliminary experiments showed that reducing the curing time below 4 min decreased neonicotinoid retention levels. For a curing time of 5 min, a TMP of 10 bar resulted in higher pollutant retention and adsorption similar to those obtained with longer curing times and higher TMP. Therefore, 5 min was the best balance between permeability and pollutant retention, combined with a TMP of 10 bar.

**Figure 1:**
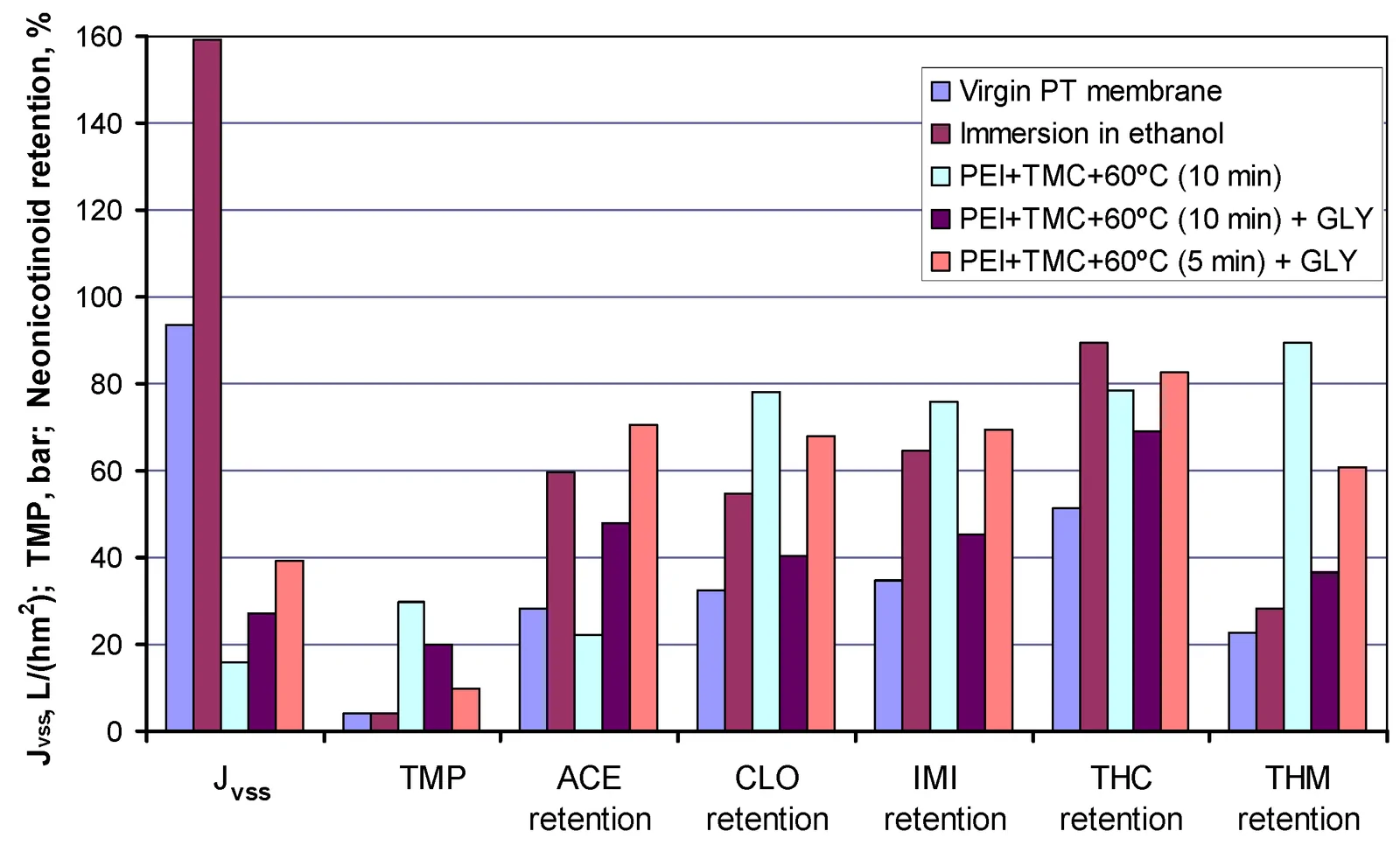
Summary of the most favorable techniques for modifying PT membranes: permeability, TMP, and neonicotinoid retention percentages.

As a summary of all the studies carried out in UP water, Figure 1 presents the most favorable results obtained after different surface modifications of the PT membrane (both by immersion in ethanol and by interfacial polymerization), as well as under different conditions and variables used (results shown in Table 5, Table 6, Tables S4 and S5). As shown, polymerization with PEI+TMC+60 °C+GLY with a 5 min curing time and operating at 10 bars results in neonicotinoid retention values between 60% and 83%, while maintaining an adequate permeate flow rate at steady state. The PEI+TMC+60 °C experiment, in the absence of GLY, was measured at VRF 1.1 because of its very low permeability, which is a disadvantage for the process. Therefore, we chose the PEI+TMC+60 °C+GLY configuration for the next stage of the study.

### 3.3. Removal of Neonicotinoids Using Modified UF Membranes by Interfacial Polymerization in Real Water Matrices

**Figure 2:**
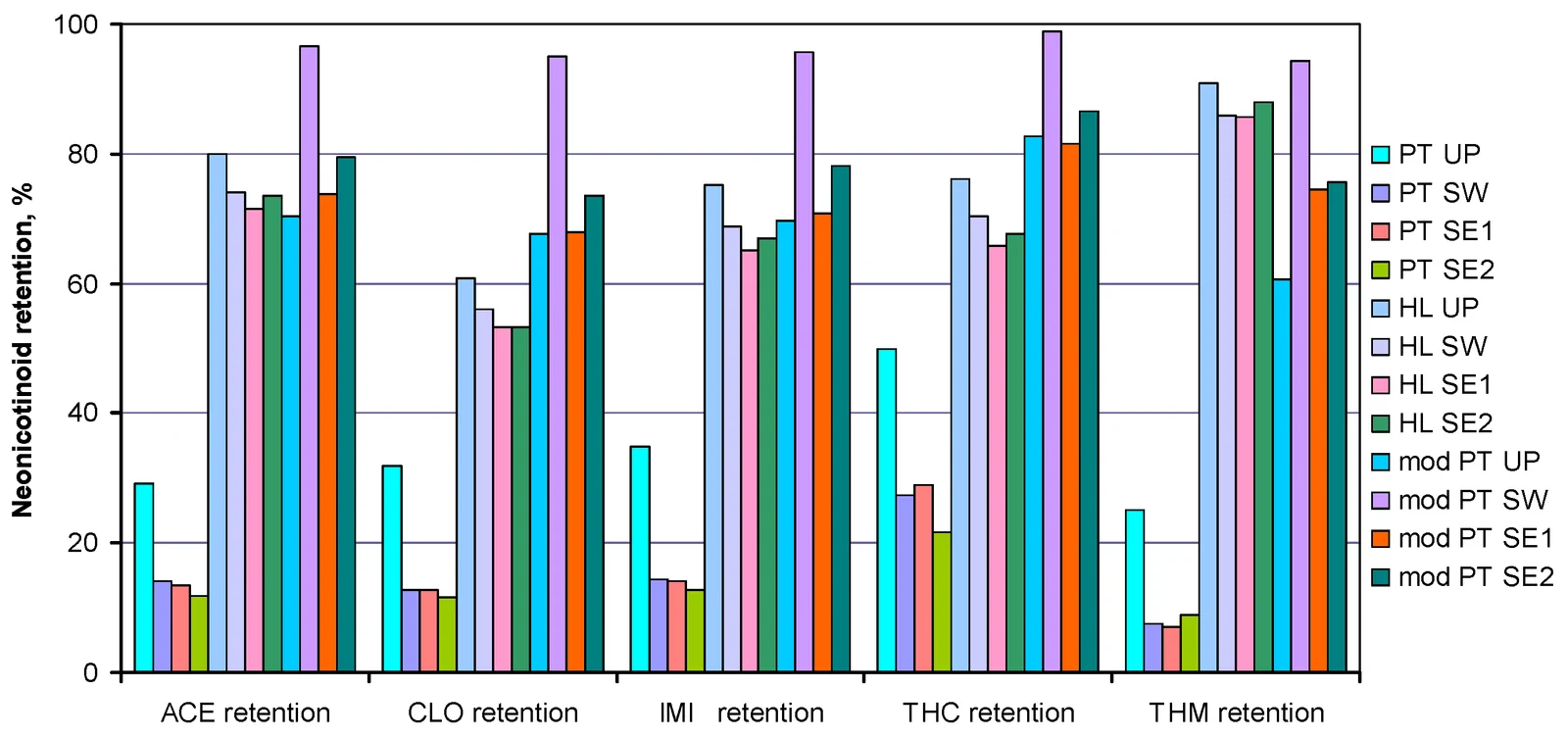
Neonicotinoid retention percentages obtained during filtration of real water matrices with virgin PT and HL membranes, and modified PT membranes by interfacial polymerization using PEI+TMC+60 °C+GLY.

After establishing the performance of modified UF membranes using different techniques in UP water, the most suitable modification conditions (polymerization with PEI, TMC, heat at 60 °C, and immersion in GLY solution) were selected for use in real water matrices. According to the conclusions reached in the previous section, PEI and TMC were applied only to the active layer using an agitated cell (9 min for PEI and 30 s for TMC), the curing time at 60 °C was 5 min, and the chosen pressure was 10 bar, seeking a balance between high levels of permeability and retention of neonicotinoids. Table 7 shows the results obtained with this modified membrane using the water matrices under study. The retention percentages were calculated both at a VRF of 3 and for the total permeate mass extracted up to that point (200 mL). The permeate flux obtained with real water matrices was lower than that with UP water. Moreover, the J_vss_/J_w1_ values were slightly lower than 1, indicating membrane fouling during the filtration of real water matrices, although the membrane recovery was very close to 100%, meaning that the fouling was predominantly external [21]. Neonicotinoid retentions were, in all cases, significantly higher than those achieved with the virgin PT membrane (see Table S2 for experiments performed at 4 bar), and even slightly higher than those obtained with the NF HL membrane. This trend is clear when comparing retention percentages at VRF 3 for virgin PT and HL membranes (Table S2) and the modified PT membrane (Table 7). While the average retention of the five neonicotinoids was around 35% in UP water and 15% in real water matrices with the virgin PT membrane, and 70–80% with the HL membrane, an average retention of 70–95% was obtained with the modified PT membrane. In addition, neonicotinoid retentions were higher in real water matrices than in UP water with the modified PT membrane, especially in the SW matrix, with retentions exceeding 94% for all compounds. These higher retentions could be attributed to the formation of an extra layer of organic matter on the membrane surface acting as a second filtration barrier, which is also consistent with the lower permeate flow observed. In addition, organic matter and inorganic ions (i.e., carbonate) present in real water matrices could contribute to concentration polarization. The retention percentages for the total permeate mass (200 mL) were significantly higher than those measured at VRF 3 because neonicotinoid concentration in the retentate increased as VRF increased, reducing the specific retention percentages measured from the initial pollutant concentration. To compare retention percentages across the entire permeate mass extracted, Figure 2 shows overall retention values for the same experiments listed in Table S2 (virgin PT and HL membranes) and Table 7 (modified PT membranes). The results clearly show the low performance of the virgin PT membrane, with values below 30% in almost all experiments, except for the retention of the more favorable neonicotinoid (THC), and the high performance of the modified membrane, which was at the level of the HL membrane, or even better, for most pollutants. Removal of each neonicotinoid in the permeate stream was almost always above 80%, reaching around 90% in SW experiments. Therefore, both membranes, modified PT and HL, can efficiently meet the required retention levels and can be considered a possible quaternary treatment option. When qualitatively comparing the energy efficiency of both alternatives, the TMP of the modified PT membrane is half that of the HL membrane, while the operating times are longer, resulting in similar energy-efficiency outcomes.

According to Table 7 and Table S2, adsorption levels were only slightly higher in the modified PT membrane than in the virgin, although retention levels were much higher. This again indicates that the modified membrane behaves more like an NF membrane and that adsorption was not the primary mechanism for neonicotinoid retention, thus facilitating continuous operation. These adsorption levels of the modified PT membrane were lower in real water matrices than in UP water, likely due to organic matter competing with neonicotinoids for adsorption, but clearly higher than those observed with the HL membrane.

**Figure 3:**
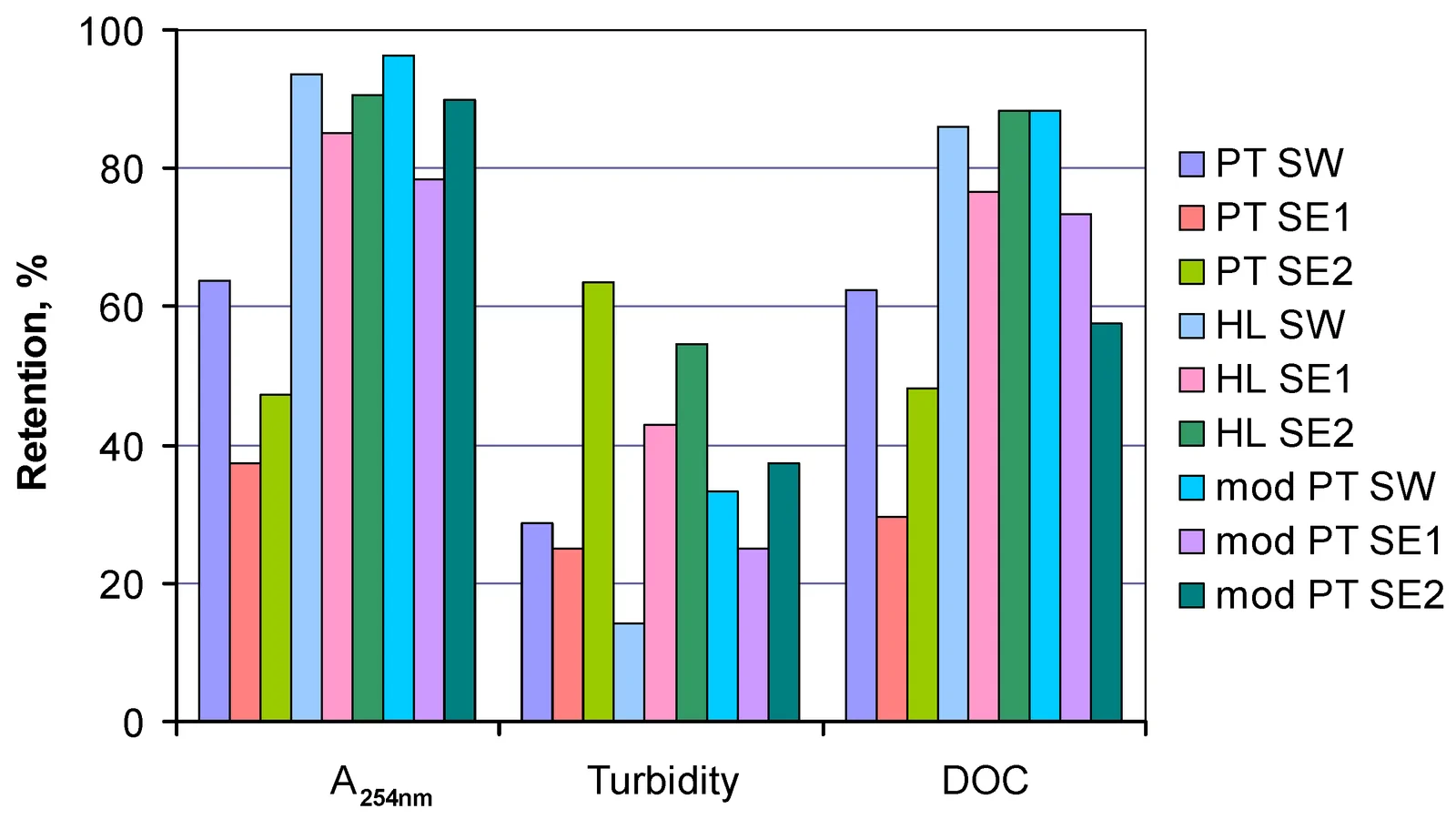
Retention percentages of water pollution parameters (A_254nm_, turbidity, and DOC) during filtration of real water matrices with virgin PT and HL membranes, and modified PT membranes by polymerization using PEI+TMC+60 °C+GLY.

Water quality parameters were analyzed, and the corresponding retention values are presented in Figure 3, along with those achieved with the virgin PT and HL membranes (see also Table S3). The modified membrane reduced both A_254nm_ and DOC considerably, reaching levels similar to those of the HL membrane. Specifically, A_254nm_ removal ranged from 78% to 96%. Turbidity reductions were more modest, remaining below 40% and lower than those achieved with the HL membrane. Meanwhile, DOC retentions ranged from 57% to 88%, indicating that the organic load in the water matrix was also reduced satisfactorily.

## 4. Conclusions

This study investigated the performance of various commercial UF and NF membranes, as well as the UF PT membrane modified using immersion and interfacial polymerization techniques. Neonicotinoid retention percentages with commercial UF membranes, such as MW, PT, and GK, were very low, with adsorption on the membrane surface being the primary retention mechanism for these micropollutants. Only the NF HL membrane achieved high retention levels, mainly through size exclusion. Increasing the pH from 6 to 9 slightly decreased neonicotinoid retention by UF membranes without affecting permeability. Retention decreased more markedly for the HL membrane, suggesting the use of a near-neutral pH. The decrease in retention observed in experiments performed with real water matrices (SW, SE1, and SE2) was significant for the UF PT membrane and very slight for the NF HL membrane.

Several surface modification techniques were applied to the PT membrane because it performed better among the UF membranes. Immersion modification of the PT membrane was promising only with ethanol solutions, with clear increases in neonicotinoid retention and permeability due to the removal of preservatives from the membrane surface and swelling of the outermost membrane layer. Conversely, the high levels of adsorption on the membrane, which was the main retention mechanism, make it difficult to use for long filtration periods.

Surface modification by interfacial polymerization techniques enabled the production of membranes with a surface layer that significantly outperformed the retention capacity of commercial PT membranes, bringing them to a range similar to that of an NF membrane. Consequently, permeability decreased, so a higher TMP was required. Among the multiple reagents, operations, conditions, and operating times tested, the most favorable configuration in terms of retention, permeability, and adsorption levels was the sequence of stages PEI+TMC+60 °C+GLY with an oven curing time of 5 min, followed by immersion in a 15% GLY solution for 4 h and subsequent operation at a TMP of 10 bar.

The final tests using this configuration with real water samples enabled a comparison of the modified membranes’ performance with that of the previously evaluated commercial PT and HL membranes. Improvements in neonicotinoid retention levels were observed in these water samples when using the modified PT membrane, reaching 80–95% retention, even higher than the HL membrane, and using a lower TMP. These results make this method a promising alternative to commercial membranes and could meet the EU removal-percentage requirements for micropollutants in WWTPs via quaternary treatment. However, to consider these membranes as a practical alternative, these conclusions should still be validated with adequate analysis and characterization of the modified membrane and with pilot plant-scale experiments at more realistic contaminant concentrations.

## Figures and Tables

**Table 1:** Physico-chemical properties of selected neonicotinoids.

Neonicotinoid	Chemical Structure	MW, g/mol	log K_OW_^1^	Charge at pH 6–9	V_m_^1^, cm^3^
Acetamiprid (ACE) C_10_H_11_ClN_4_	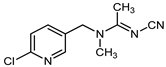	222.68	0.62	neutral	189.6
Clothianidin (CLO) C_6_H_8_ClN_5_O_2_S	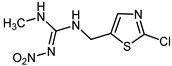	249.68	0.40	neutral	147.8
Imidacloprid (IMI) C_9_H_10_ClN_5_O_2_	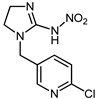	255.67	−0.43	neutral	160.1
Thiacloprid (THC) C_10_H_9_ClN_4_S	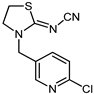	252.73	0.55	neutral	177.4
Thiamethoxam (THM) C_8_H_10_ClN_5_O_3_S	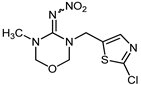	291.71	−1.16	neutral	170.2

^1^ Octanol-water distribution coefficient (log K_OW_) and molar volume (V_m_) have been determined with the software ACD/ChemSketch.

**Table 2:** Properties of target membranes.

Membrane	Material ^1^	MWCO, Da	pH	Contact Angle, °	PWP, L/h/m^2^/bar
MW	PAN	50,000	2–9	-	134.5 ± 4.3
PT	PES	5000	2–11	52.8 ± 2	24.3 ± 2.3
GK	TF	2000	2–11	44 ± 3	3.9 ± 0.4
HL	TF	150–300	3–9	30 ± 3	8.8 ± 0.6

^1^ PAN: Polyacrylonitrile; PES: Polyethersulfone; TF: Polysulfone-polyamide thin-film composite.

**Table 3:** Quality parameters of the selected water matrices.

Parameter	SW	SE1	SE2
pH	7.3	8.1	7.8
Conductivity (μS/cm)	122	804	672
A_254nm_ (cm^−1^)	0.236	0.087	0.167
DOC (mg/L)	7.9	4.7	9.2
COD (mg/L O_2_)	15	11	22
Alkalinity (mg/L CaCO_3_)	15.3	210	183
Nitrate (mg/L NO_3_^−^)	0.2	22.3	21.6
Nitrite (μg/L NO_2_^−^)	6	277	31
Ammonium (mg/L NH_3_^+^)	0.16	0.20	0.19
Total nitrogen (mg/L N)	0.4	4.2	5.6
Total phosphorus (mg/L P)	0.07	1.06	0.26
Turbidity (NTU)	0.3	0.3	0.6

**Table 4:** Results from membrane filtration experiments performed with UP water.

		MW	PT	GK	HL
MWCO, Da		50,000	5000	2000	150–300
TMP, bar		2	4	6	20
J_w1_, L/(h m^2^)		269.0	95.2	23.7	177.6
J_vss_, L/(h m^2^)		256.5	97.6	24.9	186.6
J_w2_, L/(h m^2^)		275.7	102.0	25.9	185.2
J_vss_/J_w1_		0.95	1.03	1.05	1.05
Membrane recovery, %		103	107	109	104
PWP, L/(h m^2^ bar)		134.5	23.8	3.9	8.9
Retention at VRF 3, %	ACE	5.5	29.3	11.8	79.9
	CLO	6.9	31.8	15.5	60.9
	IMI	7.3	34.7	14.2	75.1
	THC	7.1	49.9	18.1	76.0
	THM	6.8	25.1	13.4	90.8
Adsorption, %	ACE	8.1	30.5	12.9	11.9
	CLO	8.3	33.0	14.7	12.7
	IMI	9.1	35.3	14.5	13.8
	THC	9.2	57.4	19.2	18.6
	THM	8.8	26.2	13.2	10.3

**Table 5:** Results from membrane filtration experiments performed with UP water using PT membranes modified by immersion.

		Blank	Hot Water		1 M NaOH		Abs. Ethanol		60% Ethanol
Storage Time in Cold Water			1 Day	7 Days	1 Day	7 Days	1 Day	7 Days	1 Day
TMP, bar		4	4	4	4	4	4	4	4
J_w1_, L/(h m^2^)		94.0	112.0	99.9	148.6	141.0	161.7	157.1	164.9
J_vss_, L/(h m^2^)		93.6	110.6	100.9	150.8	140.1	159.1	156.1	164.8
J_w2_, L/(h m^2^)		103.0	119.6	104.4	161.9	142.4	165.9	163.0	172.9
J_vss_/J_w1_		1.00	0.99	1.01	1.01	0.99	0.98	0.99	1.00
Membrane recovery, %		110%	107%	105%	109%	101%	103%	104%	105%
PWP, L/(h m^2^ bar)		23.5	28.0	25.0	37.1	35.3	40.4	39.3	41.2
Retention at VRF 3, %	ACE	28.3	11.3	11.2	12.2	12.9	59.7	44.4	45.8
	CLO	32.6	12.3	12.6	12.7	14.1	54.9	44.0	43.9
	IMI	34.7	12.3	12.3	11.4	13.7	64.6	50.5	54.4
	THC	51.3	16.0	19.7	21.4	19.1	89.3	76.5	78.2
	THM	22.6	10.3	9.5	10.9	12.2	28.4	28.9	21.8
Adsorption, %	ACE	29.4	16.1	16.5	18.0	18.1	61.9	50.2	27.2
	CLO	34.0	18.6	19.8	20.9	21.7	59.9	50.4	50.5
	IMI	35.8	18.7	19.4	19.7	20.8	66.4	56.2	58.2
	THC	55.3	33.0	32.7	36.4	32.6	87.6	76.7	78.1
	THM	26.3	9.7	9.9	12.3	14.8	31.4	32.8	27.2

**Table 6:** Results from experiments performed with PT membranes modified by interfacial polymerization using PEI+TMC+60 °C. Influence of glycerol (15% *w*/*w*) immersion post-treatment and ethanol immersion pre-treatment.

		Blank	PEI+TMC+60 °C	PEI+TMC+60 °C+ GLY (4 h)	Ethanol (24 h)+ PEI+TMC+60 °C
TMP, bar		4	30	20	8
J_w1_, L/(h m^2^)		94.0	16.0	26.6	65.0
J_vss_, L/(h m^2^)		93.6	14.4	27.1	25.5
J_w2_, L/(h m^2^)		103.0	14.0	24.8	24.5
J_vss_/J_w1_		1.00	0.91	1.02	0.39
Membrane recovery, %		110%	87%	93%	38%
PWP, L/(h m^2^ bar)		23.5	0.53	1.33	8.1
Operation time, min		45	92	163	177
Retention at VRF 1.1, %	ACE	28.7	78.0	79.5	82.8
	CLO	34.8	75.7	76.5	82.8
	IMI	37.7	78.6	83.2	84.8
	THC	77.6	89.4	94.0	95.6
	THM	17.5	75.1	65.0	74.3
Retention at VRF 3, %	ACE	28.3	-	47.9	55.5
	CLO	32.6	-	40.3	53.0
	IMI	34.7	-	45.1	54.3
	THC	51.3	-	68.9	70.5
	THM	22.5	-	36.5	46.2
Adsorption, %	ACE	29.4	-	34.7	34.1
	CLO	34.0	-	29.4	25.9
	IMI	35.8	-	64.6	73.6
	THC	55.3	-	56.1	47.2
	THM	26.3	-	19.4	19.8

**Table 7:** Results obtained in the filtration experiments performed with the PT membrane modified by interfacial polymerization using PEI+TMC+60 °C+GLY with several water matrices.

		UP	SW	SE1	SE2
TMP, bar		10	10	10	10
J_w1_, L/(h m^2^)		39.8	21.1	22.1	27.9
J_vss_, L/(h m^2^)		39.3	20.6	18.8	25.5
J_w2_, L/(h m^2^)		37.1	21.0	21.9	27.4
J_vss_/J_w1_		0.99	0.98	0.85	0.92
Membrane recovery, %		93%	100%	99%	98%
PWP, L/(h m^2^ bar)		4.0	2.1	2.2	2.8
Retention at VRF 3, %	ACE	70.4	96.6	73.7	79.5
	CLO	67.8	94.9	67.8	73.7
	IMI	69.7	95.6	70.9	78.2
	THC	82.6	99.0	81.6	86.5
	THM	60.7	94.3	74.6	75.7
Overall retention in 200 mL of permeate, %	ACE	84.7 ± 3	98.2 ± 1	83.5 ± 2	87.7 ± 2
	CLO	84.1 ± 3	97.5 ± 2	80.3 ± 3	83.8 ± 3
	IMI	85.9 ± 2	97.5 ± 2	83.4 ± 4	88.2 ± 2
	THC	95.2 ± 2	99.5 ± 1	89.0 ± 2	92.5 ± 1
	THM	79.3 ± 4	96.4 ± 2	84.0 ± 3	84.7 ± 2
Adsorption, %	ACE	34.4	30.9	28.6	26.3
	CLO	30.3	23.0	18.5	21.4
	IMI	88.2	22.7	20.7	23.4
	THC	51.7	42.2	42.9	37.4
	THM	28.4	19.1	34.1	15.6

## Data Availability

The original contributions presented in this study are included in the article. For further inquiries, contact the corresponding authors.

## Supplementary Materials

The following supporting information can be downloaded at https://www.mdpi.com/article/10.3390/membranes16090289/s1. Text S1: Analysis of the neonicotinoids and water quality parameters; Table S1: results from membrane filtration experiments using UP water at varying pH level; Table S2: results from membrane filtration experiments performed with several water matrices; Table S3: removal percentages of water pollution parameters obtained from filtration experiments performed with several water matrices; Table S4: results from membrane filtration experiments performed with UP water using PT membranes modified by interfacial polymerization. Influence of the monomer used; Table S5: results from experiments performed with PT membranes modified by interfacial polymerization using PEI+TMC+60 °C+GLY. Effect of curing time at 60 °C and TMP.
