# Supplementary material for: Improved Removal of Neonicotinoid Insecticides from Real Water Matrices by Modified UF and NF Membranes
Source: Membranes (Basel). 2026 Aug 28;16(9):289. doi: 10.3390/membranes16090289 (PMC13609286; doi:10.3390/membranes16090289)
Supplement: Supplementary file 1 [file membranes-16-00289-s001.zip › membranes-4473098-supplementary.pdf]

Supporting information for

Improved removal of neonicotinoid insecticides from real water matrices by modified UF and NF membranes

Francisco J. Real*, Juan L. Acero, Esther Matamoros and Carolina Godoy
Departamento de Ingeniería Química y Química Física, Instituto Universitario de Investigación del Agua, Cambio Climático y Sostenibilidad (IACYS), Facultad de Ciencias, Universidad de Extremadura, Avda. de Elvas s/n, 06006-Badajoz, Spain.

*Author to whom correspondence should be addressed. e-mail: fjreal@unex.es Tel. number: +34 924489950

Table of contents

Text S1	Analysis of neonicotinoids and water quality parameters	p. S2
Table S1	Results from membrane filtration experiments using UP water at varying pH levels.	p. S4
Table S2	Results from membrane filtration experiments performed with several water matrices.	p. S5
Table S3	Removal percentages of water pollution parameters obtained from filtration experiments performed with several water matrices.	p. S6
Table S4	Results from membrane filtration experiments performed with UP water using PT membranes modified by interfacial polymerization. Influence of the monomer used.	p. S6
Table S5	Results from experiments performed with PT membranes modified by interfacial polymerization using PEI+TMC+60 °C+GLY. Effect of curing time at 60 °C and TMP.	p. S7

Text S1. Analysis of neonicotinoids and water quality parameters.

The concentration of each neonicotinoid was determined by HPLC, using a Waters Chromatograph (Alliance 2695) equipped with a 2998 Photodiode Array Detector and a Phenomenex Kinetex® C18 Column (5µm, 150mm×4.6mm). The analytical method was adapted from previous works [23,24,27]. The mobile phase consisted of a mixture of methanol and 10 mM o-phosphoric acid aqueous solution, with a flow rate of 1 mL min^{-1}, and the injection volume was 100 µL. The mixtures of the five neonicotinoids were analyzed in gradient mode. Methanol (A) and 10 mM o-phosphoric acid aqueous solution (B) percentages in gradient mode were the following:

Time (min)	A (%)	B (%)
0	25	75
3.5	25	75
8.5	45	55
12	45	55
14	25	75
18	25	75

The selected detection wavelengths were 253 nm for THM, 280 nm for IMI, 267 nm for CLO, and 236 nm for ACE and THC. The limit of quantification of the neonicotinoids was 0.02 µM and the limit of detection 0.006 µM. Chromatographic analysis began with blank samples (ultrapure water) to confirm the absence of contamination from the LC system. Variation of concentration of neonicotinoids in duplicate analyses was below 3%.

Some physico-chemical characteristics of secondary effluents were determined according to the Standard Methods [28]. Electrical conductivity (EC) and pH were measured using a

Hanna HI255 apparatus (Smithfield, RI, USA). Chemical oxygen demand (COD), total nitrogen, nitrate, ammonium, and total phosphorus concentrations were determined using Hanna analytical kits (Smithfield, RI, USA). Dissolved organic carbon (DOC) was analyzed on a TOC-multi N-C 3100 Analytic Jena device (Jena, Germany). Alkalinity was determined by acid-base titration using a 0.1 N HCl solution. All spectrophotometric measurements, including absorbance at 254 nm (A_{254nm}), were performed on a Thermo Scientific Evolution 300 apparatus (Waltham, MA, USA). Turbidity was measured using a Hanna HI98703 turbidimeter (Smithfield, RI, USA).

Table S1. Results from membrane filtration experiments using UP water at varying pH levels.

		PT			HL		
pH		6	7	9	6	7	9
TMP, bar		4	4	4	20	20	20
J_{w1}, L/(h m^2)		95.2	93.8	89.0	177.6	180.7	172.1
J_{vss}, L/(h m^2)		97.6	92.7	89.3	186.6	174.6	170.9
J_{w2}, L/(h m^2)		102.0	102.3	94.4	185.2	175.4	176.3
J_{vss}/J_{w1}		1.03	0.99	1.00	1.05	0.97	0.99
Membrane recovery, %		107%	109%	111%	104%	97%	102%
PWP, L/(h m^2 bar)		23.8	23.4	22.3	8.9	9.0	8.6
Retention at VRF 3, %	ACE	29.3	23.5	24.8	79.9	77.0	60.0
	CLO	31.8	25.8	25.4	60.9	60.3	51.5
	IMI	34.7	28.5	28.6	75.1	72.6	58.3
	THC	49.9	42.4	46.9	76.0	74.7	60.8
	THM	25.1	19.2	14.9	90.8	85.6	69.5
Adsorption, %	ACE	30.5	26.6	26.8	11.9	15.8	9.4
	CLO	33.0	26.7	28.3	12.7	16.8	16.3
	IMI	35.3	30.1	31.3	13.8	17.2	15.7
	THC	57.4	49.2	53.7	18.6	21.8	20.4
	THM	26.2	22.1	17.3	10.3	14.5	13.1

Table S2. Results from membrane filtration experiments performed with several water matrices.

		PT				HL			
**Water matrix**		**UP**	**SW**	**SE1**	**SE2**	**UP**	**SW**	**SE1**	**SE2**
TMP, bar		4	4	4	4	20	20	20	20
J_{w1}, L/(h m^2)		95.2	87.6	90.1	95.3	177.6	184.5	172.3	189.1
J_{vss}, L/(h m^2)		97.6	84.1	84.2	90.6	186.6	168.2	150.9	168.8
J_{w2}, L/(h m^2)		102.0	87.6	86.6	99.5	185.2	175.4	172.1	172.4
J_{vss}/J_{w1}		1.03	0.99	0.93	0.95	1.05	0.91	0.88	0.89
Membrane recovery, %		107	103	96	104	104	95	100	91
PWP, L/(h m^2 bar)		23.8	21.3	22.5	23.8	8.9	9.2	8.6	9.5
Retention at VRF 3, %	ACE	29.3	14.1	13.4	11.8	79.9	74.1	71.6	73.6
	CLO	31.8	12.8	12.7	11.6	60.9	56.0	53.4	53.4
	IMI	34.7	14.3	14.0	12.7	75.1	68.9	65.1	67.0
	THC	49.9	27.3	29.0	21.6	76.0	70.3	65.8	67.7
	THM	25.1	7.6	7.0	8.9	90.8	86.0	85.7	87.8
Overall retention in 200 mL of permeate, %	ACE	31.2	23.8	24.0	21.5	87.5	82.9	81.4	82.5
	CLO	33.2	24.5	24.9	22.6	74.8	70.1	68.3	69.3
	IMI	35.4	26.5	27.7	24.9	84.9	79.5	77.5	78.9
	THC	59.3	49.0	50.9	43.9	86.9	81.8	79.8	81.3
	THM	26.2	12.8	13.0	13.0	94.3	90.6	90.7	91.6
Adsorption, %	ACE	30.5	20.1	19.7	18.0	11.9	16.1	19.6	11.1
	CLO	33.0	20.8	20.6	19.0	12.7	15.6	18.7	12.4
	IMI	35.3	22.1	22.9	20.8	13.8	16.8	19.9	11.9
	THC	57.4	39.1	40.8	34.8	18.6	21.8	29.1	18.2
	THM	26.2	11.6	11.2	11.7	10.3	14.7	16.8	9.7

Table S3. Removal percentages of water pollution parameters obtained from filtration experiments performed with several water matrices.

	**PT**			**HL**		
**Water matrix**	**SW**	**SE1**	**SE2**	**SW**	**SE1**	**SE2**
A_{254nm} removal, %	63.8	37.4	47.3	93.5	85.0	90.7
DOC removal, %	62.4	29.6	48.1	85.9	76.5	88.4
Total nitrogen removal, %	30.0	30.0	42.0	100	56.1	56.0
Total phosphorus removal, %	100	62.0	80.0	100	100	100
Turbidity removal, %	28.6	25.0	63.6	14.3	42.9	54.5

Table S4. Results from membrane filtration experiments performed with UP water using PT membranes modified by interfacial polymerization. Influence of the monomer used.

		**Blank**	**PEI 60 °C**	**PEI+TMC 60 °C**	**PEI+TMC 60 °C**	**PEI/SDS+TMC 60 °C**
TMP, bar		4	6	6	30	6
J_{w1}, L/(h m^2)		94.0	20.3	9.8	16.0	9.9
J_{vss}, L/(h m^2)		93.6	18.4	9.5	14.4	9.4
J_{w2}, L/(h m^2)		103.0	16.4	9.2	14.0	9.9
J_{vss}/J_{w1}		1.00	0.91	0.97	0.91	0.94
Membrane recovery, %		110%	81%	94%	87%	99%
PWP, L/(h m^2 bar)		23.5	3.4	1.64	0.53	1.65
Operation time, min		46	61	71	92	76
Retention at VRF 1.1, %	ACE	28.7	64.7	90.7	78.0	80.9
	CLO	34.8	62.1	91.7	75.7	79.0
	IMI	37.7	70.6	93.2	78.6	81.9
	THC	77.6	94.3	98.3	89.4	93.7
	THM	17.5	28.6	87.1	75.1	75.0

SDS: Sodium dodecyl sulphate surfactant

Table S5. Results from experiments performed with PT membranes modified by interfacial polymerization using PEI+TMC+60 °C+GLY. Effect of curing time at 60 °C and TMP.

		**Blank**	**8 min**	**8 min**	**5 min**	**5 min**
TMP, bar		4	15	10	8	10
J_{w1}, L/(h m^2)		94.0	36.4	18.0	36.1	39.8
J_{vss}, L/(h m^2)		93.6	36.3	16.2	35.0	39.3
J_{w2}, L/(h m^2)		103.0	37.3	16.1	30.5	37.1
J_{vss}/J_{w1}		1.00	1.00	0.90	0.97	0.99
Membrane recovery, %		110%	102%	90%	85%	93%
PWP, L/(h m^2 bar)		23.5	2.43	1.81	4.51	3.98
Operation time, min		45	122	279	131	114
Retention at VRF 1.1, %	ACE	28.7	92.1	90.2	82.4	94.7
	CLO	34.8	92.2	91.5	82.0	95.3
	IMI	37.7	92.7	95.1	84.3	96.9
	THC	77.6	97.1	100	92.5	100
	THM	17.5	85.1	74.9	73.3	87.0
Retention at VRF 3, %	ACE	28.3	72.6	60.9	54.1	70.4
	CLO	32.6	69.9	54.3	50.4	67.8
	IMI	34.7	71.6	57.9	54.0	69.6
	THC	51.3	83.9	82.4	70.8	82.6
	THM	22.5	64.1	48.6	43.8	60.7
Adsorption, %	ACE	29.4	29.8	47.0	31.1	34.3
	CLO	34.0	30.1	38.9	31.2	30.2
	IMI	35.8	85.4	78.9	73.7	88.2
	THC	55.3	43.8	67.0	44.4	51.7
	THM	26.3	22.3	29.6	23.1	28.4

References

23. Real, F.J.; Acero, J.L.; Benitez F.J.; Matamoros, E. Elimination of Neonicotinoids by Ozone-Based Advanced Oxidation Processes: Kinetics and Performance in Real Water Matrices. *Sep. Purif. Technol.* **2022**, *301*, 121975. [https://doi.org/10.1016/j.seppur.2022.121975]

24. Real, F.J.; Acero, J.L.; Matamoros, E. Removal of Neonicotinoids Present in Secondary Effluents by Ferrate(VI)-Based Oxidation Processes. *Environ. Sci. Pollut. Res.* **2024**, *31*, 29684-29694. [https://doi.org/10.1007/s11356-024-33167-3]

27. Acero, J.L.; Real, F.J.; Benitez, F.J.; Matamoros, E. Degradation of Neonicotinoids by UV Irradiation: Kinetics and Effect of Real Water Constituents. *Sep. Purif. Technol.* **2019**, *211*, 218-226. [https://doi.org/10.1016/j.seppur.2018.09.076]

28. Clesceri, L.S.; Greenberg, A.E.; Trussell, R.R. Standard Methods for the Examination of Water and Wastewater, 17th edn., APHA, AWWA, WPCF, Washington, DC. **1989**. ISBN 9780875531618.
